# Editorial: Towards a mechanistic understanding of depression, anxiety, and their comorbidity: perspectives from cognitive neuroscience

**DOI:** 10.3389/fnbeh.2023.1268156

**Published:** 2023-08-15

**Authors:** Masaru Tanaka, Chong Chen

**Affiliations:** ^1^Danube Neuroscience Research Laboratory, ELKH-SZTE Neuroscience Research Group, Eötvös Loránd Research Network, University of Szeged (ELKH-SZTE), Szeged, Hungary; ^2^Division of Neuropsychiatry, Department of Neuroscience, Yamaguchi University Graduate School of Medicine, Yamaguchi, Japan

**Keywords:** depression, anxiety, emotions, fear, schizophrenia, comorbidity, neuromodulation, interleukins

Depression and anxiety are two prevalent mental health disorders that have a significant impact on individuals' lives and society (Brenes, 2007; Malone and Wachholtz, 2018). These conditions can occur independently or co-occur, leading to a complex and challenging clinical picture (Chen, 2022; Lei et al., 2022; Battaglia et al., 2023b; Di Gregorio and Battaglia, 2023). To shed light on the underlying mechanisms and potential treatment targets for these disorders, cognitive neuroscience offers valuable insights (Ravache et al., 2023). The Research Topic, “*Towards a mechanistic understanding of depression, anxiety, and their comorbidity: perspectives from cognitive neuroscience*,” delves into exploring the common background and current challenges related to depression, anxiety, and their comorbidity from a cognitive neuroscience perspective (Garofalo et al., 2017, 2019; Borgomaneri et al., 2023; Turrini et al., 2023).^1^ This Research Topic aims to comprehensively explore the distinct and shared mechanisms involved in depression and anxiety to identify potential therapeutic targets and personalized clinical approaches (Battaglia et al., 2023a; Battaglia M. R. et al., 2023). Understanding the cognitive neuroscience aspects of these disorders is essential for preventive measures and early interventions, reducing their impact on individuals and society (Wojtalik et al., 2018). The articles delve into emotional processing, shedding light on areas such as flexible regulation of emotional expression, vicarious fear-learning, and reactive action inhibition. Integration of insights from attachment theory and cognitive neuroscience is highlighted to enhance understanding of the comorbidity between depression and anxiety. The relationship between interoceptive fearfulness and comorbid anxiety and depression is another significant focus. Furthermore, the role of gene-stressor interactions in emotional processing and their connection to depression is thoroughly explored.

Three articles focus on the topic of anxiety and depression and their comorbidity. They explore the relationship between emotional regulation and psychopathological symptoms, increased interoceptive fearfulness and reactivity in comorbid anxiety and depression, and the integration of insights from attachment theory and cognitive neuroscience to better understand these mental health conditions. Uncertainty regarding the interrelationships between suppressed abilities, affective disorders or psychopathology symptoms, cognitive involvement, and their dependence on the neural circuitry is one of the most stressful issues (Anderson et al., 2019). Gonzalez-Escamilla et al. explore the relationship between flexible emotional regulation and mental health and show that both enhancing and suppressing abilities are associated with resilience, depression, and anxiety. This study provides valuable insights into the relationship between emotional regulation and mental health, shedding light on the importance of being able to adaptively express and regulate emotions. There is a need for more longitudinal studies to determine the cause-and-effect relationship between anxiety and depression, which is one of the current challenges in the field (Kalin, 2020). Ironside et al. explores the increased interoceptive fearfulness and reactivity in individuals with both anxiety and depression compared to those with depression only. This study provides valuable insights into the unique face of comorbid anxiety and depression, specifically in terms of increased interoceptive fearfulness and reactivity, and highlights the need for further investigation into the mechanisms underlying this relationship. Comorbid depression and anxiety require integrated and individualized approaches considering biological, psychological, and social factors (Remes et al., 2021). Rajkumar integrates insights from attachment theory and cognitive neuroscience and show that insecure attachment may contribute to the development of both depression and anxiety, via a variety of cognitive neuroscience pathways. The integration of perspectives and findings from attachment theory and cognitive neuroscience is of more than theoretical interest, as it has the potential to lead to improved strategies for prevention, early intervention, and more effective treatment of this pattern of comorbidity.

Three articles focus on the neural mechanisms underlying emotional processing and reactive action inhibition. They explore the influence of vicarious fear-learning on reactive action inhibition, reactive action inhibition when facing valence-independent emotional stimuli, and sex differences in amygdalohippocampal oscillations and neuronal activation in a rodent anxiety model. Understanding the influence of vicarious fear learning on reactive action inhibition and its potential repercussions on mental health are among the current challenges (Sun et al., 2023). Battaglia et al.(a) report that vicarious fear learning can have a critical impact on cognitive abilities, making a neutral image as threatening as innate negative stimuli, and can impact behavioral control. The study provides insights into the impact of vicarious fear-learning on cognitive abilities and its potential implications for mental health. Investigating how emotions affect our ability to control actions in response to emotional stimuli remains a current challenge (Tyng et al., 2017). Battaglia et al.(b) explores how individuals react and inhibit their actions when faced with emotional stimuli that are not necessarily positive or negative in valence. Individuals' reactions and inhibitive actions in response to emotional stimuli are not necessarily positive or negative in valence, which can help us understand the neural mechanisms underlying deficient inhibitory control in various psychopathologies and mood disorders. Based on an enhanced comprehension of the underlying neural mechanisms, a current challenge involves formulating more efficient and personalized treatments for anxiety disorders (Craske et al., 2023). Vila-Merkle et al. sheds light on the underlying neural mechanisms of anxiety and show that male and female rodents demonstrate distinct neural oscillations in response to an anxiogenic drug. The study offers valuable information on the neural mechanisms of anxiety and sex differences in rodent models and calls for more in-depth investigation into the sex differences in anxiety and depression (Lei et al., 2021).

Two articles explore the relationship between mental health conditions such as obsessive-compulsive symptoms and schizophrenia, and the role of gene-stressor interactions in emotional processing and depression. The development of effective treatment strategies for schizophrenia patients with comorbid obsessive-compulsive symptoms, especially those who are resistant to therapy, is one of the current challenges (Zink, 2014; Zhao et al., 2022). the influence of disorganized symptoms, duration of schizophrenia, and drug resistance on the manifestation of obsessive-compulsive symptoms. Panov and Panova examine the influence of disorganized symptoms, schizophrenia duration, and drug resistance on obsessive-compulsive symptom manifestation in schizophrenia patients. The study highlights the necessity for further research and tailored treatment approaches for patients with comorbid conditions. Identifying the underlying mechanisms and potential therapeutic targets for the association between interleukin-6 rhythm, amygdala emotional hyporeactivity, and depression is one of the current challenges (Hu et al., 2022). Hakamata et al. examine the relationship between interleukin-6 rhythm, emotional hyporeactivity in the amygdala, and depression, with a focus on the role played by gene-stressor interactions in regulating these processes. Their study offers insights into these complex interactions, guiding potential therapeutic interventions and future research.

Complementing human research, animal model-based preclinical research has played a crucial role in elucidating the complex interplay of genetic, environmental, and pharmacological factors that contribute to mental illness (Tanaka and Telegdy, 2008, 2013; Telegdy et al., 2010, 2011; Tanaka et al., 2011, 2012; Palotai et al., 2014). These models mimic disease conditions, enabling the evaluation of symptomatology and potential interventions (Chu et al., 2023; Polyák et al., 2023; Tanaka et al., 2023b). Valuable insights into disease mechanisms, treatment testing, and therapeutic efficacy, as well as structural changes and imaging techniques for clinical applications, are provided (Garofalo et al., 2021; Nyatega et al., 2022; Okanda Nyatega et al., 2022; Adamu et al., 2023; Chen et al., 2023; Du et al., 2023; Kim et al., 2023; Liu M. et al., 2023; Liu N. et al., 2023; Rymaszewska et al., 2023; Tanaka et al., 2023a; Zhou et al., 2023). The combination of preclinical and clinical research contributes to the development of innovative therapeutics and personalized medicine (Balogh et al., 2021; Senevirathne et al., 2023; Tajti et al., 2023).

In conclusion, we hope that this Research Topic serves as a pivotal platform for investigating the neural underpinnings of depression and anxiety. By delving into the cognitive neuroscience perspective, researchers aim to unlock new avenues for tailored treatment strategies and preventive measures, ultimately improving the lives of those affected by these challenging mental health disorders. As we advance our knowledge of the mechanisms behind depression and anxiety, we move closer to a future where individuals can receive personalized care and support to overcome these burdensome conditions. We extend our gratitude to all the authors who contributed to this Research Topic and express our appreciation to the reviewers for their valuable feedback. We eagerly anticipate future contributions that will further advance the field of depression, anxiety, and their comorbidity. Your support and dedication play a crucial role in shaping the progress and potential of this rapidly growing field.

## Author contributions

MT: Conceptualization, Writing—original draft, Writing—review and editing. CC: Conceptualization, Writing—review and editing.

## Footnotes

^1^ https://academic.oup.com/book/39433/chapter/339164773
